# Siamese masked reconstruction with temporal rotation consistency for inertial-based human activity recognition

**DOI:** 10.1038/s41598-026-64846-5

**Published:** 2026-08-14

**Authors:** Francisco M. Calatrava-Nicolás, Todor Stoyanov, Oscar Martinez Mozos

**Affiliations:** 1 Department of Science and Technology, Örebro University, Örebro, 70182 Sweden; 2 Human Robotics, DFESTS, University of Alicante, Alicante, 03690 Spain; 3 MAD-Robotics, Escuela Técnica Superior de Ingeniería y Diseño Industrial (ETSIDI), Universidad Politécnica de Madrid, Madrid, 28012 Spain

**Keywords:** Engineering, Mathematics and computing

## Abstract

Inertial-based Human Activity Recognition (HAR) aims at inferring activities performed by an individual from data on wearable inertial sensors. However, HAR often suffers from poor generalization to unseen users due to data heterogeneity across different individuals and conditions. While collecting and curating large labeled datasets could help alleviate this limitation, it is also costly. To reduce this gap without relying on extensive annotated data, we propose a self-supervised framework grounded in how IMU signals transform under rotation: applying a rotation matrix changes how motion projects onto the sensor axes without altering the underlying motion. A model trained to align representations of a signal and its rotated counterpart is therefore encouraged to capture motion content rather than sensor-frame patterns. We exploit this property by training a Siamese masked convolutional autoencoder that learns by reconstructing masked inputs while aligning representations of original signals and its rotated version, further regularized by a temporal consistency loss that enforces agreement between the temporal structure of original and rotated representations within each window. We evaluate on four public HAR benchmarks covering diverse scenarios, including activities of the daily living (ADL) and sports activities, using cross-subject evaluation, where our method yields improvements of +1.4 and +1.2 percentage points over the strongest baseline with linear and MLP probes respectively, averaged across datasets and sensor positions. We further show that our method obtains competitive results against the fully supervised baseline in low-data regimes. The code is available on https://github.com/FranciscoCalatrava/Rotation_Siamese_Masked.git.

## Introduction

Inertial-based Human Activity Recognition (HAR) aims to infer activities performed by an individual from accelerometer and gyroscope signals measured on the body^1^. These inertial signals are typically acquired using an Inertial Measurement Unit (IMU), which provides both linear acceleration and angular velocity. Together, these two cues offer a compact yet expressive representation of human motion dynamics, capturing translational movements as well as changes in orientation and rotation. Moreover, IMUs are widely available in everyday devices such as smartwatches, bands, and wearable nodes^2–4^. This availability enables inertial HAR in a broad spectrum of applications, ranging from everyday well-being and lifestyle monitoring^5,6^ to clinical assessment^7^ and human–robot interaction^8^ among others. In these contexts, HAR systems enable the analysis of complex human behaviors and generate data-driven insights that could support clinical decision-making, personalized health interventions, elderly care, and more adaptive interactive technologies^9–11^.

While HAR can leverage multiple sensing modalities, we focus on inertial sensing due to its favourable trade-off between information content, energy consumption, and hardware cost. Moreover, inertial sensing is a core modality in widely used public datasets^1,12–15^. Beyond combining modalities, HAR systems can also integrate information from multiple IMUs placed at different body locations to improve robustness and discriminability. However, such multi-sensor configurations increase deployment complexity (e.g., user compliance, synchronization, calibration, and battery management) and may be impractical in real-world scenarios where only one wearable device is usually available (e.g. smartwatch or smartphone). Therefore, this study considers a single-sensor, single-position setting, in which the model is trained and evaluated using data collected from the same IMU placement on the body. To analyse how sensor placement influences recognition performance under this constraint, we consider four representative positions that are common in public datasets: the right wrist, left wrist, right ankle, and left ankle.

Deep learning has greatly advanced HAR in recent years, yet supervised models trained only on activity labeled data still struggle to generalise to unseen users. This generalisation gap is largely driven by data heterogeneity arising from sources such as inter-subject variability, i.e. different individuals perform the same activity differently^16–18^. A straightforward way to reduce this gap is to collect and annotate large datasets that reflect diverse real-world conditions, but this is expensive and time-consuming^19^. Consequently, recent work has explored learning strategies that improve generalisation without relying on large-scale manual annotation, for instance by introducing auxiliary tasks to support the primary HAR objective^17,18,20,21^.

A particular class of auxiliary tasks are those that require no additional labels, since they can exploit information from large-scale unlabeled sensor data beyond the activity labels. In this context, self-supervised learning (SSL) has become a popular strategy in HAR, where the model is trained to solve a proxy task defined directly from the sensor signals (e.g., predicting masked segments^22^, reconstructing inputs^23,24^, or relating transformed versions of the same window^25,26^). These proxy objectives encourage the model to capture intrinsic structure in the data, yielding representations that can be transferred to the downstream activity recognition task.

Using geometric transformations as a training signal is particularly appealing in self-supervised learning because applying them to an input data sample (i.e., image, inertial signal, etc.) creates multiple transformed versions (or “views”) of the same sample without requiring labels, enabling the model to learn meaningful representations by identifying and extracting the consistent underlying information shared across these different views. This idea has been widely explored in computer vision, where different views of an image are used to learn representations that remain consistent across transformations^27,28^. In HAR, a similar principle applies, but the meaning of a transformation is different compared to the image domain: inertial signals are governed by the sensor reference frame and the underlying measurement physics rather than visual appearance. Nevertheless, transformations in HAR are typically used as data augmentations, without explicitly leveraging the fact that the applied transformation is known and can therefore impose principled constraints on how IMU measurements should relate across views. This creates a gap between using transformations to diversify training data and using them as explicit supervision for representation learning. In this work, we focus on 3-dimensional rotations to bridge this gap. Physically, a 3-dimensional rotation deterministically maps accelerometer and gyroscope signals across axes via a rotation matrix^29^. We choose rotations because they are grounded in sensor geometry: they correspond to a change of reference frame that preserves the underlying motion while re-expressing it across axes. In contrast, many commonly used augmentations (e.g., jitter, scaling, time-warping, cropping) can alter the temporal dynamics of the signal in ways that are not uniquely tied to a geometrically interpretable sensor transformation. In addition to this, the rotation transformation has been shown to be an effective IMU augmentation, improving robustness^30^. We therefore posit that rotations can provide informative self-supervision because they preserve the temporal dynamics of a movement while redistributing that information across sensor axes.

Building on this, we introduce a rotation-aware pretraining objective that uses sensor-frame rotations as a structured source of self-supervision. Concretely, given an inertial window, we generate multiple views of the same window, which the framework exploits through three coupled self-supervised objectives: (i) masked reconstruction^24^ objective, (ii) a modified version of siamese objective^27^ that aligns representations from rotated and original signals, and (iii) a constraint that encourages rotation-consistent temporal structure between the representations of original and rotated views. Overall, the framework combines masked reconstruction with rotation-derived constraints that inject additional self-supervision during pretraining.

We evaluate our approach on four well-established HAR datasets comprising a diverse set of activities, including activities of daily living (ADL) and sports activities: the Physical Activity Monitoring (PAMAP2) dataset^31^, the Mobile Health (MHEALTH) dataset^13^, the REAListic sensor DISPlacement (REALDISP)^15^, and the Wearable and Egocentric Activity Recognition (WEAR) dataset^14^. We use subject-wise folds to expose the network to inter-subject variability, running each fold for 5 different random seeds under 4 different body locations (left/right wrist and left/right ankle). Our approach yields more informative representations than the baselines, with improvements of +1.4 and +1.2 percentage points over the strongest competitor with linear and MLP heads respectively, averaged over datasets and body positions. We also perform an analysis under different labeled data regimes in which our method obtains competitive results against the fully supervised baseline. In addition, we offer an ablation study in which we compare the performance of our framework to better understand the contributions of each of the parts. Finally, we present an activity-wise analysis together with a symmetry analysis to better characterise the proposed method.

## Related works

Early deep learning approaches typically formulate sensor-based Human Activity Recognition (HAR) as a supervised classification problem over fixed-length windows of inertial signals. Within this setting, convolutional models learn local temporal patterns directly from raw streams^32^, while recurrent architectures such as LSTMs model longer temporal dependencies^33^. Hybrid CNN–LSTM designs combine both mechanisms to capture complementary spatial/temporal structure^34^. Attention-based methods further improve performance by emphasizing informative sensor channels or devices^35^. More recently, transformer-based models have been introduced to strengthen cross-sensor fusion and capture long-range dynamics efficiently^36^. Despite substantial improvements in recognition accuracy, supervised HAR methods often require large labeled datasets and may generalise poorly under realistic deployment conditions. In particular, performance can drop due to data distribution shifts. In inertial-based HAR, this shift is produced by the way different individuals implement the same activity (inter-person variability), the sensor position (placement variability), and hardware differences between sensors^18,19,37^, among others. These factors are especially relevant in wearable scenarios, causing a generalization gap when these models are tested under these data distribution shifts.

Although collecting large-scale datasets could address this generalization gap, doing so is also costly^19^. This motivates the development of methods that narrow this gap while reducing dependence on manual annotation. In this context, self-supervised learning (SSL) has emerged as a promising alternative for HAR. By leveraging unlabeled data samples through proxy objectives, SSL can learn transferable representations that approach and in some cases exceed supervised baselines while using substantially fewer labelled samples for downstream training^1^. SSL methods for HAR exploit a variety of proxy objectives to learn from unlabeled data, including transformation recognition^25^, temporal predictive coding^22,38^, contrastive view agreement^26,39^, non-contrastive view agreement^27,28^, and masked reconstruction^23,24,40^ among others. In this work we focus on two families that directly underpin our approach: non-contrastive view-based methods, which learn from augmented signal pairs, and reconstruction-based methods, which learn by recovering masked portions of the input.

On the reconstruction side, early work applies autoencoders to inertial signals, training an encoder-decoder to recover the full input from its latent representation^41^. However, this objective risks producing trivial representations, as the encoder may learn to compress the signal without capturing meaningful structure^42^. Introducing masking addresses this by forcing the model to infer missing portions from context, yielding richer representations. This idea has been applied to inertial HAR using both transformer-based^23,40^ and convolutional^24^ backbones. Related developments in computer vision, such as ConvNeXt V2 and VideoMAC, also show that masked autoencoding can be combined with convolutional encoders, while requiring attention to the interaction between masking and convolutional architectures^43,44^. In contrast to these image/video works, our focus is not on convolutional architecture design, but on using a convolutional backbone for inertial HAR and complementing masked reconstruction with rotation-consistency objectives grounded in IMU geometry. Within view-based methods, early work proposes predicting whether a signal has been augmented, under the assumption that this encourages robust representations^25^. More recent non-contrastive approaches adapt ideas from computer vision. In particular, BYOL^28^ aligns representations of two differently augmented views using two branches: an online branch that predicts a stable representation produced by a target branch, encouraging invariance to the chosen augmentations. Building on this line, Siamese learning can be also adapted to HAR^27^, training two symmetric branches to agree on embeddings produced from two augmented views, showing improved results over BYOL in the original evaluation. Despite their effectiveness, prior self-supervised approaches for inertial-based HAR leave two gaps that motivate our work. First, in inertial-based HAR, reconstruction-based and view-based objectives are often explored separately, leaving open whether a unified objective could yield richer representations. Second, in the case of view-based approaches, not all augmentations are equally informative as a self-supervised signal. Among common choices, rotations have been shown to be an effective augmentation in SSL pipelines for inertial HAR^26^. Beyond empirical effectiveness, rotations have a notable geometric grounding: unlike other transformations (.i.e. jitter or scaling), they correspond to a deterministic reference-frame change that preserves motion dynamics while redistributing signal energy across axes in an algebraically consistent way, a structure that carries richer self-supervisory potential than a generic perturbation. Yet existing methods normally exploit rotations as a data diversification tool, reducing them to a source of invariance and leaving this potential unexploited.

In this work, we propose a hybrid approach that combines a modified version of the Siamese-style view alignment^27^ with masked reconstruction, aiming to strengthen the self-supervised signal by jointly leveraging invariance learning and structure-preserving reconstruction. To ground the multi-view setting in the geometric structure identified above, we instantiate the multi-view setting using rotated versions of the same signal. Additionally, we introduce a time-level agreement term that explicitly exploits the structured relationship induced by rotations across time-resolved latent representations, turning rotation from a data diversification tool into a direct source of structured self-supervision that complements the global agreement imposed by the Siamese term.

## Method

In this section, we present our proposed method for HAR using a single inertial sensor. We first introduce our rotation augmentation protocol. We further consider a two-stage learning setting. In the pretraining stage, we learn an encoder from unlabeled inertial windows using a self-supervised objective. In the down-stream stage, we evaluate the pretrained encoder on a labeled HAR dataset by training a lightweight classifier on top of the frozen representations.

**Fig. 1:**
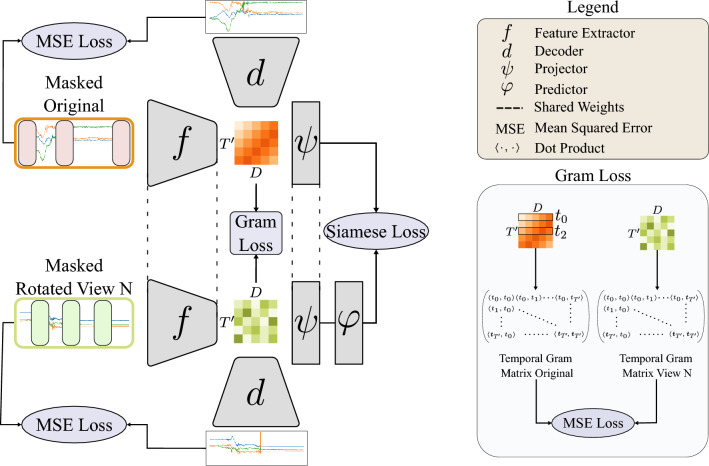
General overview of the proposed method. Given an inertial window (accelerometer + gyroscope), we sample *K* random rotations to generate rotated views. The original window and each rotated view are then independently masked by hiding subsets of their temporal segments, and the resulting masked windows are encoded by *f* into latent representations. A Siamese loss encourages each rotated-view representation to align with that of the original signal. To additionally preserve temporal motion structure, a temporal Gram loss matches the time–time similarity matrices of the features extracted from the original and rotated signals, enforcing consistency in their pairwise temporal relationships. Finally, a decoder is trained with an MSE reconstruction loss to reconstruct the masked portions of each input window. The network is trained end-to-end by jointly optimizing the three losses.

The overall framework is illustrated in Fig. 1 and comprises four modules: an encoder *f(· )*, a decoder *d(· )*, a projection head $\psi (\cdot )$, and a prediction head $\varphi (\cdot )$. Details of the network architectures are provided in the supplementary material (Appendix B). The encoder *f(· )* maps an input window, in which a random subset of time steps has been zeroed out (masked), to a latent representation, which a decoder *d(· )* then uses to reconstruct the missing measurements.

For the consistency objectives, we first map the encoder features through a projection head $\psi (\cdot )$, producing the projected embeddings from which a temporal self-similarity matrix is computed. The intuition is that the relational structure between time steps should remain invariant under rotation; this objective, therefore, enforces consistency between the similarity matrix of the original window and that of its rotation-augmented counterpart. Complementarily, we define a modified Siamese loss using an additional prediction head $\varphi (\cdot )$, which takes the projected embedding of the rotated view and predicts the projected embedding of the original view, thereby encouraging the rotated representation to align with the original one^27^. We detail each step in the following sections.

### Data augmentation

Prior work has shown that rotation augmentation, i.e., applying a 3-dimensional rotation (via a rotation matrix) to inertial measurements at each time step, is particularly effective for diversifying training samples in HAR^26,30^. Beyond its empirical effectiveness, rotations are also well grounded in sensor geometry: a 3-dimensional rotation corresponds to a change of the IMU reference frame, i.e., a different sensor orientation at the same body location, that preserves the underlying motion and its temporal structure, while linearly re-expressing accelerometer and gyroscope signals across axes. Building on this property, we treat rotations not merely as a data diversification strategy but as an explicit source of supervisory signal, incorporating them as the basis for two self-supervised consistency objectives described in the followings sections.

Formally, let $\mathcal {D}_{u}=\{x_i\}_{i=1}^{N}$ denote the unlabeled dataset used for self-supervised pretraining, where each window $x_i \in \mathbb {R}^{C \times T}$ contains $C\in \{3,6\}$ channels (tri-axial accelerometer and, when available, tri-axial gyroscope) over *T* time steps. For each sample $x_i$, we construct a set of *K* rotated views

1 $$\begin{aligned} \mathcal {V}(x_i) = \left\{ x_i,\ \tilde{x}_i^{(1)},\ \ldots ,\ \tilde{x}_i^{(K)} \right\} , \end{aligned}$$

where $\tilde{x}_i^{(k)}$ denotes the *k*-th rotated view of $x_i$.

To generate $\tilde{x}_i^{(k)}$, we sample an axis–angle rotation by drawing a random unit axis $u^{(k)} \in \mathbb {R}^{3}$ and an angle $\theta ^{(k)} \sim \mathcal {U}(-\alpha _{\max }, \alpha _{\max })$. The corresponding rotation matrix $R^{(k)} \in SO(3)$ is computed via Rodrigues’ formula^45^. Let $a_i(t)\in \mathbb {R}^{3}$ and $g_i(t)\in \mathbb {R}^{3}$ denote the accelerometer and gyroscope vectors of $x_i$ at time step *t*, respectively. We apply the same rotation to both modalities at every time step:

2 $$\begin{aligned} \tilde{a}_i^{(k)}(t) = R^{(k)} a_i(t), \qquad \tilde{g}_i^{(k)}(t) = R^{(k)} g_i(t), \qquad t=1,\ldots ,T, \end{aligned}$$

and define the rotated window as $\tilde{x}_i^{(k)} = [\tilde{a}_i^{(k)};\tilde{g}_i^{(k)}] \in \mathbb {R}^{6 \times T}$.

### Self-supervised pretraining objective

Our self-supervised objective combines three complementary losses: (i) a masked reconstruction loss applied to each view, (ii) a Siamese inspired consistency loss^27^ between the original and rotated views, and (iii) a temporal self-similarity loss that enforces structural consistency across views at the time-step level. Each loss is described in detail in the following subsections.

#### Masked reconstruction task

We follow the masked reconstruction strategy similar to^24,46^, where the mask is sampled at the temporal resolution induced by the encoder downsampling, but applied to the raw input signal before the encoder forward pass. Specifically, since the encoder *f(· )* downsamples the temporal dimension from *T* to *T'*, we independently sample a binary mask $m'_{c} \in \{0,1\}^{T'}$ for each channel *c ∈ C*, setting each temporal position in *T'* to 0 (masked) with probability *ρ =0.3* and 1 (visible) with probability *1-ρ =0.7*. The resulting mask $m' \in \{0,1\}^{C \times T'}$ is then upsampled to the input resolution via nearest-neighbor interpolation for reconstruction, obtaining the mask $m \in \{0,1\}^{C \times T}$. This sampling procedure is applied independently to each sample in $\mathcal {V}(x_i)$, yielding a distinct mask $m_i$ for the original sample and $m_i^{(k)}$ for each rotated view $\tilde{x}^{(k)}$. We then apply each mask via element-wise multiplication and process the masked input through the encoder *f(· )*, which means that the weights are shared over the original and rotated views. For the original sample, this reads:

3 $$\begin{aligned} h_i = f( x_i \odot m_i) \in \mathbb {R}^{D \times T'} \end{aligned}$$

where *D* is the feature dimension, *T'* the downsampled temporal dimension, and *⊙* the element-wise product. The same operation applies to each rotated view, yielding $h_i^{(k)} = f(\tilde{x}_i^{(k)} \odot m_i^{(k)})$.

We then decode each latent representation back to the input space via the decoder *d(· )*. For the original sample:

4 $$\begin{aligned} \hat{x}_i = d(h_i) \in \mathbb {R}^{C \times T} \end{aligned}$$

and analogously $\hat{x}_i^{(k)} = d(h_i^{(k)})$ for each rotated view.

The reconstruction loss compares the input window $x_i \in \mathbb {R}^{C\times T}$ with its reconstruction $\hat{x_i}$, and is evaluated only on masked positions. Let $m_i \in \{0,1\}^{C \times T}$ be the binary mask, where $m_{i,(c,t)}=0$ indicates a masked position. We define the set of masked positions as

5 $$\begin{aligned} \mathcal {I}_{\textrm{mask}}(m_i)\triangleq \{\, (c,t) \in \{1,\dots ,C\} \times \{1,\dots ,T\} \;:\; m_{i,(c,t)} = 0 \,\}. \end{aligned}$$

The masked reconstruction loss is then defined as

6 $$\begin{aligned} \mathcal {L}_{\textrm{mask}}(x_i,\hat{x_i};m_i)= \frac{1}{|\mathcal {I}_{\textrm{mask}}(m_i)|} \sum _{(c,t) \in \mathcal {I}_{\textrm{mask}}(m_i)} \left( x_{i,(c,t)} - \hat{x}_{i,(c,t)} \right) ^2 . \end{aligned}$$

We apply the same objective to the original sample and its *K* rotated views, and average:

7 $$\begin{aligned} \mathcal {L}_{\textrm{rec}}= \frac{1}{K+1}\left( \mathcal {L}_{\textrm{mask}}(x_i,\hat{x}_{i};m_i) +\sum _{k=1}^{K}\mathcal {L}_{\textrm{mask}}(\tilde{x}_{i}^{(k)},\hat{x}_{i}^{(k)};m_{i}^{(k)}) \right) . \end{aligned}$$

Since the reconstruction loss is evaluated independently for each view using its corresponding mask, only the masked positions of that same view contribute to the loss (Eq. 6). Consequently, even though masks are sampled independently across original and rotated views, visible regions in one view do not directly supervise the reconstruction of masked raw signal values in another view.

#### Siamese consistency loss

To encourage rotation-consistent representations, we use a similarity loss inspired by the Siamese objective^27^. The main intuition of this loss is to reduce the discrepancy between the representations of an original signal and its rotated counterpart. To this end, we use a projector $\psi (\cdot )$ and a predictor $\varphi (\cdot )$. The original signal and its rotated views follow asymmetric paths. The original signal with latent representation $h_i$ is processed only by the projector:

8 $$\begin{aligned} u_i = \psi (h_i), \qquad \bar{u}_i = \textrm{pool}(u_i) \end{aligned}$$

where $\textrm{pool}(\cdot )$ denotes average pooling over *T'*. Each rotated view with latent $h_i^{(k)}$ is processed by both the projector and the predictor:

9 $$\begin{aligned} u_i^{(k)} = \psi (h_i^{(k)}), \qquad p^{(k)}_i = \varphi (u_i^{(k)} ), \qquad \bar{p}^{(k)}_i = \textrm{pool}(p^{(k)}_i) \end{aligned}$$

Finally, we minimize the cosine distance between the projection of the original signal ($\bar{u}_i$) and the output of the prediction for the different rotated views ($\bar{p}^{(k)}_i$)

10 $$\begin{aligned} \mathcal {L}_{\textrm{siam}} = \frac{1}{K}\sum _{k=1}^{K} \left( 1 - \cos \!\left( \bar{p}^{(k)}_i, \text {sg}(\bar{u}_i)\right) \right) , \end{aligned}$$

where $\text {sg}(\cdot )$ denotes the stop-gradient operator which prevents the gradient flow for the original signal projection and *K* refers to the number of views. This loss regularizes the model by encouraging the representations of rotated views to remain close to the representation of the original signal. Unlike the standard Siamese objective, which enforces bidirectional alignment between two augmented views of the same signal, we use a one-sided objective in which we treat the original signal as a fixed anchor, guiding the representations of rotated views toward it.

#### Time-level agreement via temporal gram loss

The embedding agreement introduced in the previous subsection encourages global similarity between the representations of the rotated views and the original signal. However, since it relies on temporal pooling, it does not explicitly retain fine-grained temporal structure. To better match the temporal dynamics of the original signal and its rotated versions, we introduce a time-level agreement loss based on a temporal Gram matrix^47^.

**Fig. 2:**
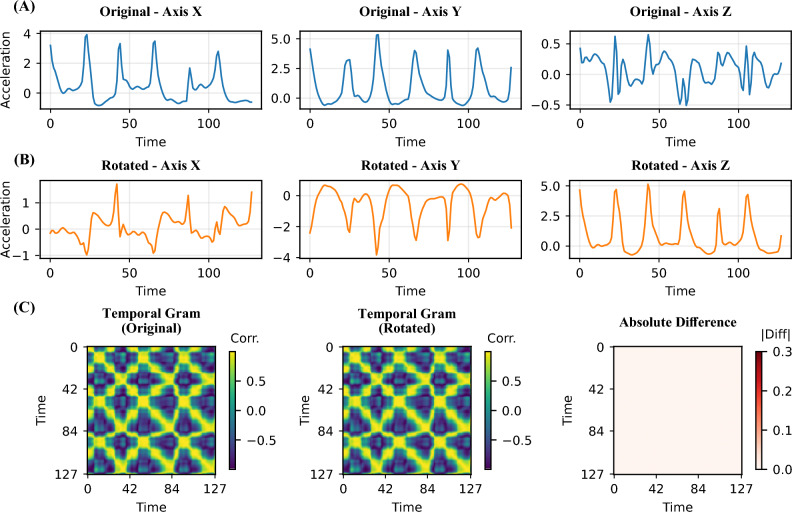
Rotation preserves temporal structure in raw inertial signals but requires explicit enforcement in learned representations. (**A**) Original 3-axis accelerometer signal exhibiting periodic motion patterns. (**B**) The same signal after applying a random 3D rotation—individual axes appear completely different, with information redistributed across spatial dimensions. (**C**) Temporal Gram matrices, which capture time-to-time correlation patterns, remain nearly identical between original and rotated signals, demonstrating that rotation preserves temporal dynamics at the raw signal level.

The key insight of this loss objective is that rotation transforms inertial signals in a way that redistributes information across spatial axes while preserving temporal patterns. Although a rotated signal appears visually different from its original (see Fig. 2(A) and (B)), the underlying temporal dynamics, such as the timing of peaks or periodicity, remain intact, merely expressed in a rotated coordinate frame. Based on this intuition, we define the temporal gram matrix, which shows the similarity of each time data point with respect to all the other data points. As shown in Fig. 2 (C), the temporal gram matrix on the input space for original and rotated signals looks identical. However, this property does not necessarily carry over to neural representations without an explicit constraint, which motivates our temporal Gram loss as a way to encourage the latent space to reflect it, under the hypothesis that this can be beneficial for learning.

Let $u_i, u^{(k)}_i \in \mathbb {R}^{D \times T'}$ be the projected features for the original and *k*-th rotated view respectively. We first L2-normalize^48^ both along the feature dimension (*D*) before computing the temporal Gram matrix^47^:

11 $$\begin{aligned} G(u) = u^{\top }u \in \mathbb {R}^{T' \times T'}. \end{aligned}$$

This matrix encodes the cosine similarity between each latent temporal representation and all others, capturing the pairwise temporal structure of the learned representation. We then encourage temporal consistency between rotated and original views by minimizing:

12 $$\begin{aligned} \mathcal {L}_{\textrm{gram}} = \frac{1}{K}\sum _{k=1}^{K} \left\| G(u_i) - G(u^{(k)}_i) \right\| _F^2. \end{aligned}$$

This term is designed to enforce consistency in the pairwise temporal relationships between rotated and original views, providing additional supervision beyond the global embedding invariance of Eq. 10. In this setting, the Gram loss is computed between projected features obtained from independently masked views (see Eq. 3), and therefore relates temporal similarity structures that may be based on different visible regions.

#### Overall pretraining objective

The final self-supervised objective is a weighted sum of the three presented learning objectives:

13 $$\begin{aligned} \mathcal {L}_{\textrm{ssl}} = \lambda _{\textrm{rec}}\mathcal {L}_{\textrm{rec}} +\lambda _{\textrm{siam}}\mathcal {L}_{\textrm{siam}} +\lambda _{\textrm{gram}}\mathcal {L}_{\textrm{gram}}. \end{aligned}$$

where $\lambda _{rec}$, $\lambda _{siam}$, and $\lambda _{gram}$ are weights that helps to balanced the training. Finally, the full pre-training process is described in Algorithm 1.

**Algorithm 1:**
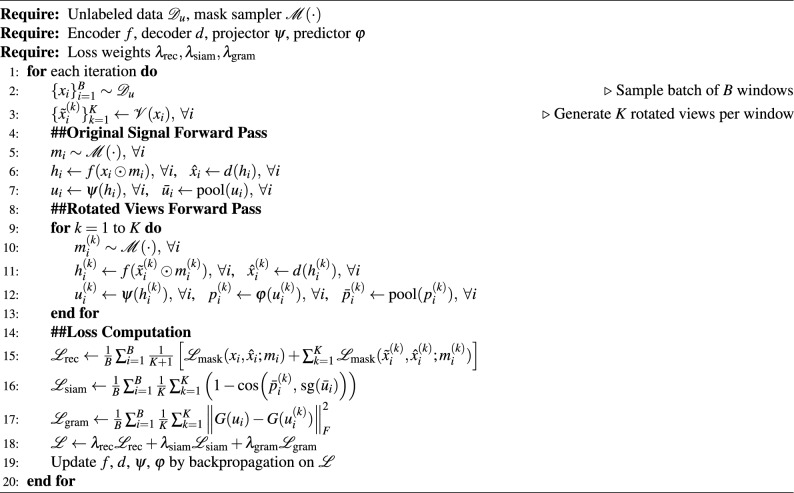
Self-supervised pre-training.

## Experimental setup

This section contains all the details needed for reproducibility of the experiments. First we present the datasets used in the study together with the evaluation protocol. Second, we explain the baselines that we defined for the comparison of our method. Finally, we describe the hyperparameters used in this study.

We evaluate our method on four well established public HAR datasets: PAMAP2^49^, MHEALTH^13^, REALDISP^15^, and WEAR^14^. PAMAP2^49^ comprises 54 channels from IMUs on the dominant wrist, chest, and leg of 9 subjects performing 18 activities (12 activities from a protocol and 6 additional activities). In this study we use the activities corresponding to the protocol. In addition, we omit subject 9 due to insufficient samples. MHEALTH^13^ comprises 12 activities of 10 participants who wore the IMU on the chest, right wrist, and left ankle. REALDISP^15^ includes 33 sport activities carried out by 17 participants while using 9 body-worn sensors on different body parts (left calf, left thigh, right calf, right thigh, back, left lower arm, left upper arm, right lower arm, right upper arm). WEAR^14^ includes 18 sport activities from 22 participants using 4 body-worn accelerometers (both wrists and both ankles).

For all datasets, we cleaned NaN values using a last-value strategy, falling back to next-value when no preceding value was available. We used the data without any further preprocessing other than resampling to 50 Hz where necessary. We segment the signals using a 128-sample sliding window with 50% overlap (i.e. 2.56 seconds per window). Each window is labelled according to the majority class among all time steps within that window. Across all datasets, we use only the accelerometer and gyroscope signals from the wrist and ankle positions, as these are the body locations available in all four datasets, ensuring a consistent input configuration of *C=6* channels throughout all experiments. Two exceptions apply: for REALDISP, we use the lower arm as a proxy for the wrist and the calf as a proxy for the ankle, as these are the closest available positions to the target body locations; for WEAR, only accelerometer signals are available, so gyroscope channels (consequently, *C=3*). The details for the activities considered for each dataset are available in supplementary material (Appendix A Table 2).

To assess the impact of inter-subject variability while keeping the computational cost tractable, we adopt a subject-wise cross-fold validation protocol. For each dataset, we partition the participants into disjoint groups of *N* subjects, ensuring that no individual appears in more than one group. In each fold, one group is held out for testing. From the remaining groups, one group is used for validation and the rest of the groups for training. Crucially, pre-training is performed using only the training fold subjects, ensuring that no information from validation or test subjects leaks into the learned representations. This procedure is repeated until each group has served as the test set once. We set *N=2* for PAMAP2 and MHEALTH, *N=4* for REALDISP, and *N=5* for WEAR, resulting in a number of folds ranging from 4 to 5. The exact distribution of participants per fold is shown in the supplementary material (Appendix A Table 1). Finally, to account for randomness and ensure fair comparisons across methods, each experiment is repeated with five random seeds (42, 43, 44, 45, and 46). We then report the mean and standard deviation of the subject-wise cross-validation results across seeds, using Macro F1-score as the evaluation metric^50^.

We compare our method against a supervised baseline and several self-supervised baselines. Specifically, the baselines are selected to evaluate three aspects: the benefit of self-supervised pre-training over supervised learning from scratch, the contribution of rotation-based augmentation as a pretext signal, and the effect of combining masked reconstruction with consistency-based objectives. Specifically, the baselines are:

**Supervised baseline (from scratch)**: We randomly initialize the encoder *f(· )* and train the encoder and classification head end-to-end using labeled data only. This baseline serves as a reference for the performance achievable without any pre-training, allowing us to assess the benefit of self-supervised pre-training over direct supervised learning from scratch.

**Single-task transformation prediction**^25^: We train the encoder with a single proxy task that performs binary classification of whether an input window has been transformed by a rotation or not. This is a single-task variant of the multi-task framework proposed in^25^, and serves as a baseline that uses the same rotation augmentation as our method but as a discriminative self-supervised task rather than a self-supervised consistency objective.

**Siamese**^27^: A Siamese baseline using the same encoder architecture as ours, where rotated versions of the same window are used as augmented views. The model is trained using the original Siamese objective, enforcing bidirectional alignment between views which belong to the same original window.

**Masked Convolutional Autoencoder (MCAE)**^24^: We adopt a masked reconstruction proxy task using our encoder backbone, in which parts of the input signal are masked and the model is trained to reconstruct the missing portions from the remaining observed content.

All frameworks were re-implemented from scratch. To ensure a fair comparison, all self-supervised methods were trained under identical data settings, using the same fold splits and the same augmentation policy applied to the original data. For all self-supervised baselines and our method, the encoder weights are frozen after pre-training and a classification head is trained on top using labeled data only. We evaluate two head configurations: a linear layer and a shallow MLP, in order to assess the quality of the learned representations under both linear and non-linear evaluation protocols. Additionally, all experiments use the same encoder architecture (network definition available in the supplementary material Appendix B Table 3) to isolate the effect of the self-supervised pretext task proposed in our methodology.

All experiments were conducted on an HPC cluster equipped with heterogeneous GPUs. We used a single global hyperparameter configuration across methods, unless a prior work reported method-specific settings.

For the self-supervised pre-training stage, we used Adam with a batch size of 512 windows, a learning rate of $5\times 10^{-4}$, and weight decay of $1\times 10^{-5}$. The learning rate was reduced by a factor of 0.5 when the validation loss plateaued (patience of 5 epochs). Pre-training was run for up to 200 epochs with early stopping based on the validation loss (patience of 30 epochs). For the masking strategy, we used a masking ratio of *ρ =0.3*. For the rotation augmentation, each original window was augmented into *K=4* rotated views, with rotation angles sampled uniformly from $[-180^\circ , 180^\circ ]$ per axis. The loss weights were set to $\lambda _{\textrm{rec}}=1.0$, $\lambda _{\textrm{siam}}=0.5$, and $\lambda _{\textrm{gram}}=10.0$, selected empirically based on validation performance.

After pre-training, we trained a linear or MLP classifier (networks definition available in the supplementary material Appendix B Tables 7 and 8) on top of the frozen encoder with a batch size of 128 for up to 200 epochs, using Adam with a learning rate of $5\times 10^{-4}$ and weight decay of $1\times 10^{-5}$. We applied the same learning-rate scheduler as in pre-training and used early stopping based on the validation macro F1-score with a patience of 30 epochs. The amount of labeled data used for fine-tuning is varied across experiments and is described during the discussion of the experimental results.

## Results

This section contains the experimental results and discussion of the proposed method for four different body positions (left/right wrist and left/right ankle). First, we present the comparison of our proposed approach with the baseline methods explained in the previous section. All methods are evaluated under two different classification heads: MLP and Linear. Linear means that the classification head is modeled by a single linear layer on top of the frozen encoder features, in order to assess the linear separability of the learned representations. MLP means that the classification head is modeled by a shallow multi-layer perceptron (MLP) on top of the frozen encoder features, in order to assess whether the learned representations contain task-relevant information that may require a non-linear decision function to be extracted. To train both the linear and MLP heads, we use 100% of the available labeled data in the train set. Next, we conduct a data-efficiency study to assess the behavior of the proposed method under low-data regimes. We then present ablation studies on the self-supervised objective from Equation 13 to asses the impact of the different learning objectives in the downstream classification. Similar to the comparison with previous methods, for the fine-tuning stage we use 100% of the labeled data in the train set. Finally, we present activity-specific classification results from symmetric body parts in order to assess the position invariance of the learned representations and identify any dataset-specific biases that may affect symmetric body positions differently.

Table 1 reports Macro F1-score comparisons for wrist- and ankle-worn sensors, benchmarked against the baseline methods previously described. Our method demonstrates competitive performance across both classification heads (Linear and MLP) and all evaluated sensor placements in each dataset. Specifically, across all dataset–placement–head configurations considered (24 in total), it achieves the best average Macro F1-score in 19 cases and the second-best in the remaining four. Importantly, when results are averaged across datasets, our method consistently outperforms all SSL baselines at each of the four body positions (see the *Average* row in Table 1). When we further average the Macro F1-scores across the four body positions, the mean improvement over the closest SSL competitor is +1.4 percentage points with the linear classifier and +1.2 percentage points with the MLP classifier. Among the five remaining cases in which we do not rank first, two correspond to the combination of Siamese baseline in MHEALTH dataset with MLP classification head, indicating that some dataset-specific characteristics may favour this approach together with the MLP classifier. Additionally, the gains for our method are most pronounced when the downstream classifier is a linear layer: our method achieves the best SSL performance in 11 out of 12 configurations. This indicates that our proposed SSL task learns well-separated latent representations, so competitive performance can be obtained even with a simple linear classifier. When the evaluation head is upgraded to an MLP, the overall ranking remains favorable to our approach, but the gap between methods becomes smaller. This trend suggests that more expressive heads can partially compensate for weaker representations by learning additional non-linear decision boundaries, whereas the linear evaluation more directly reflects representation quality. Nevertheless, in all settings where we do not rank first in terms of average F1-Score Macro, we remain second among the SSL baselines, reinforcing the robustness of the proposed method across heterogeneous conditions.

**Table 1:** Performance comparison in terms of Macro F1-score (mean ± std) for right/left ankle/wrist. **Bold** and underlined indicate the best and second-best SSL methods by mean Macro F1, respectively.

		Dataset	Supervised	Self-supervised (SSL)			
			End-to-End	SingleTask	Siamese	MCAE	Ours
Right Ankle	Linear	REALDISP	0.736±0.010	0.323±0.017	0.348±0.061	0.590±0.019	**0.603±0.009**
		WEAR	0.608±0.012	0.442±0.018	0.401±0.033	0.569±0.007	**0.571±0.009**
		*Average*	0.672±0.064	0.382±0.060	0.375±0.027	0.580±0.011	**0.587±0.016**
	MLP	REALDISP	0.671±0.015	0.489±0.017	0.592±0.034	0.594±0.011	**0.603±0.005**
		WEAR	0.609±0.010	0.511±0.015	0.489±0.006	**0.587±0.013**	0.582±0.018
		*Average*	0.640±0.031	0.500±0.011	0.540±0.051	0.591±0.004	**0.593±0.011**
Left Ankle	Linear	MHEALTH	0.575±0.023	0.328±0.053	0.482±0.031	**0.559±0.019**	0.534±0.031
		PAMAP2	0.515±0.025	0.206±0.017	0.337±0.051	0.503±0.013	**0.504±0.017**
		REALDISP	0.694±0.025	0.432±0.013	0.400±0.039	0.530±0.012	**0.538±0.010**
		WEAR	0.555±0.013	0.400±0.020	0.362±0.032	0.525±0.009	**0.545±0.008**
		*Average*	0.585±0.067	0.341±0.087	0.396±0.055	0.529±0.020	**0.530±0.016**
	MLP	MHEALTH	0.602±0.024	0.391±0.026	**0.592±0.023**	0.546±0.032	0.577±0.043
		PAMAP2	0.505±0.025	0.336±0.020	0.457±0.039	**0.512±0.017**	0.509±0.020
		REALDISP	0.628±0.023	0.523±0.007	0.555±0.016	0.557±0.011	**0.565±0.011**
		WEAR	0.550±0.009	0.474±0.021	0.474±0.030	0.563±0.013	**0.572±0.006**
		*Average*	0.571±0.047	0.431±0.072	0.519±0.056	0.544±0.020	**0.556±0.027**
Right Wrist	Linear	MHEALTH	0.559±0.030	0.470±0.049	0.509±0.023	0.544±0.032	**0.594±0.024**
		PAMAP2	0.612±0.042	0.321±0.034	0.505±0.037	0.619±0.020	**0.630±0.012**
		REALDISP	0.338±0.015	0.085±0.017	0.142±0.019	0.321±0.016	**0.344±0.028**
		WEAR	0.618±0.008	0.371±0.020	0.401±0.020	0.446±0.017	**0.475±0.020**
		*Average*	0.532±0.114	0.312±0.142	0.389±0.149	0.483±0.112	**0.511±0.112**
	MLP	MHEALTH	0.544±0.039	0.494±0.048	**0.645±0.021**	0.569±0.021	0.587±0.023
		PAMAP2	0.561±0.036	0.398±0.012	0.558±0.025	0.629±0.018	**0.640±0.019**
		REALDISP	0.301±0.019	0.142±0.008	0.237±0.022	0.339±0.017	**0.372±0.020**
		WEAR	0.593±0.017	0.473±0.020	0.496±0.012	0.542±0.006	**0.544±0.005**
		*Average*	0.500±0.116	0.377±0.140	0.484±0.152	0.520±0.109	**0.536±0.101**
Left Wrist	Linear	REALDISP	0.710±0.008	0.484±0.019	0.340±0.054	0.568±0.013	**0.583±0.010**
		WEAR	0.614±0.009	0.373±0.009	0.416±0.023	0.485±0.031	**0.507±0.020**
		*Average*	0.662±0.048	0.429±0.055	0.378±0.038	0.526±0.041	**0.545±0.038**
	MLP	REALDISP	0.648±0.018	0.567±0.016	0.570±0.031	0.598±0.009	**0.613±0.006**
		WEAR	0.597±0.014	0.453±0.026	0.502±0.010	0.517±0.011	**0.537±0.008**
		*Average*	0.623±0.026	0.510±0.057	0.536±0.034	0.557±0.041	**0.575±0.038**

*Note:* The supervised baseline trains both the encoder and the classifier head end-to-end using activity labels. In contrast, for SSL we keep the encoder frozen during fine-tuning and train only the classifier head.

**Fig. 3:**
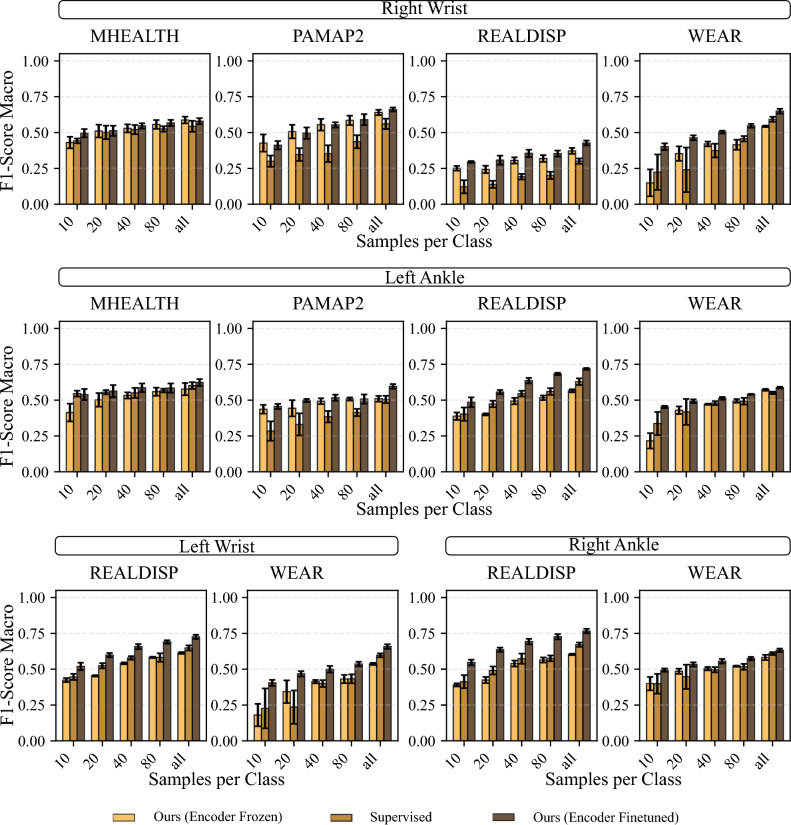
Macro F1-scores for the right/left wrist and right/left ankle across MHEALTH, PAMAP2, REALDISP, and WEAR at varying samples-per-class. We compare our method with a fully supervised baseline trained end-to-end from scratch, using the same encoder architecture and MLP classification head in both cases. For our method, we evaluate two settings: a frozen-encoder setting, in which only the classifier is trained while the encoder weights learned through the SSL task are frozen, and a fine-tuned setting, in which the encoder is further optimized on the downstream task together with a newly initialized MLP classification head. Our method remains competitive with the supervised baseline in the frozen-encoder setting and typically outperforms it in the fine-tuned setting, consistently across sample regimes.

We next study the effect of limited labeled data by defining per-activity training budgets. Figure 3 reports macro F1-score for our method and the end-to-end supervised baseline under 10, 20, 40, and 80 training windows per activity (each window is 2.56 seconds), as well as the full dataset (all). Because the supervised baseline trains the encoder and the classifier head from scratch, we report our method in two comparable variants: (i) a frozen pretrained encoder with only the classification head (MLP) trained, and (ii) fine-tuning the pretrained encoder with a newly initialized classification head (MLP). Across datasets and budgets, our method remains competitive even with a frozen encoder, particularly in low-data regimes for the right-wrist sensor. In addition, our method generally surpasses the supervised baseline when fully fine-tuned, highlighting the benefit of our self-supervised pretraining as an initialization stage prior to fine-tuning on the downstream HAR task. Moreover, our approach fully finetuned leads to clear improvements in the lowest-data regimes (10 and 20 samples per class) across sensor positions, increasing the average F1-Score macro while also generally reducing the standard deviation. This effect is most evident on the WEAR dataset, where the supervised baseline exhibits both low performance and high variance at 10 and 20 samples per class. Notably, this pronounced variability is not observed to the same extent for the corresponding sensor positions in the other datasets. A key difference is that WEAR is the only dataset in our study that provides accelerometer-only signals (no gyroscope), whereas the remaining datasets include both accelerometer and gyroscope measurements. While we do not isolate modality effects explicitly, these results suggest that the supervised baseline becomes more sensitive to low data when complementary inertial cues are missing, and that our pretraining scheme provides a more stable initialization than training from scratch, improving robustness when supervision and sensor information are limited. However, further experiments are needed to confirm this hypothesis.

Next, we present an ablation study to analyse the contribution of each component in the self-supervised objective defined in Eq. 13. Table 2 reports the results for ankle- and wrist-mounted sensors across all datasets. We compare the reconstruction-only objective $L_{rec}$, which corresponds to MCAE, a unidirectional Siamese baseline $L_{siam\_unidir}$ encourages similarity from the rotated version of a signal to the original, and three variants of our proposed framework: $L_{rec}+L_{siam}$, $L_{rec}+L_{gram}$, and the full objective $L_{rec}+L_{siam}+L_{gram}$. As in the previous experiments, the encoder is kept frozen after self-supervised pretraining and evaluated using Linear and MLP classifiers.

**Table 2:** Ablation study on right/left ankle and wrist sensors. Classification performance is reported as macro F1-score ($\textrm{mean}\pm \textrm{std}$) across runs; the best and second-best mean scores are shown in bold and underlined, respectively. $L_{rec}$ denotes the reconstruction-only variant, equivalent to MCAE; $L_{siam\_unidir}$ denotes the unidirectional Siamese objective, which enforces similarity from the rotated signal to the original; $L_{rec}+L_{siam}$ adds the Siamese similarity loss to the reconstruction loss; $L_{rec}+L_{gram}$ adds the Gram loss to the reconstruction loss; and $L_{rec}+L_{siam}+L_{gram}$ corresponds to the full proposed method.

		Dataset	Baselines		Ours		
			$L_{rec}$	$L_{siam\_unidir}$	$L_{rec}+L_{siam}$	$L_{rec}+L_{gram}$	$L_{rec}+L_{siam}+L_{gram}$
Right Ankle	Linear	REALDISP	0.590±0.019	0.375±0.037	0.579±0.019	0.596±0.015	**0.603±0.009**
		WEAR	0.569±0.007	0.388±0.021	0.568±0.008	0.563±0.009	**0.571±0.009**
		Average	0.580±0.011	0.382±0.009	0.574±0.005	0.580±0.023	**0.587±0.016**
	MLP	REALDISP	0.594±0.011	0.589±0.019	0.593±0.021	** 0.611 ± 0.021**	0.603±0.005
		WEAR	**0.587±0.013**	0.491±0.021	0.579±0.010	0.579±0.007	0.582±0.018
		Average	0.591±0.004	0.540±0.069	0.586±0.007	** 0.595 ± 0.022**	0.593±0.011
Left Ankle	Linear	MHEALTH	**0.559±0.019**	0.468±0.040	0.553±0.034	0.558±0.025	0.534±0.031
		PAMAP2	0.503±0.013	0.402±0.075	**0.509±0.018**	0.503±0.012	0.504±0.017
		REALDISP	0.530±0.012	0.294±0.041	0.529±0.006	** 0.544 ± 0.014**	0.538±0.010
		WEAR	0.525±0.009	0.351±0.041	0.520±0.014	0.520±0.036	**0.545±0.008**
		Average	0.529±0.020	0.379±0.074	0.528±0.016	** 0.531 ± 0.025**	0.530±0.016
	MLP	MHEALTH	0.546±0.032	**0.618±0.028**	0.571±0.033	0.558±0.026	0.577±0.043
		PAMAP2	0.512±0.017	0.502±0.005	0.513±0.017	**0.517±0.019**	0.509±0.020
		REALDISP	0.557±0.011	0.514±0.040	0.555±0.015	0.552±0.014	**0.565±0.011**
		WEAR	0.563±0.013	0.434±0.021	0.556±0.007	0.550±0.021	**0.572±0.006**
		Average	0.544±0.020	0.517±0.076	0.549±0.022	0.544±0.019	**0.556±0.027**
Right Wrist	Linear	MHEALTH	0.544±0.032	0.524±0.069	0.558±0.023	0.576±0.028	**0.594±0.024**
		PAMAP2	0.619±0.020	0.535±0.027	0.627±0.018	0.627±0.011	**0.630±0.012**
		REALDISP	0.321±0.016	0.185±0.039	0.335±0.020	** 0.357 ± 0.023**	0.344±0.028
		WEAR	0.446±0.017	0.324±0.049	**0.506±0.016**	0.481±0.034	0.475±0.020
		Average	0.483±0.112	0.392±0.169	0.507±0.108	0.511±0.119	**0.511±0.112**
	MLP	MHEALTH	0.569±0.021	**0.617±0.067**	0.571±0.019	0.585±0.014	0.587±0.023
		PAMAP2	0.629±0.018	0.577±0.022	0.636±0.013	0.635±0.021	**0.640±0.019**
		REALDISP	0.339±0.017	0.352±0.018	0.353±0.014	** 0.373 ± 0.014**	0.372±0.020
		WEAR	0.542±0.006	0.395±0.056	**0.549±0.008**	0.538±0.005	0.544±0.005
		Average	0.520±0.109	0.486±0.131	0.527±0.106	0.533±0.114	**0.536±0.101**
Left Wrist	Linear	REALDISP	0.568±0.013	0.285±0.117	0.570 ±0.010	**0.590±0.015**	0.583±0.010
		WEAR	0.485±0.031	0.339±0.040	0.503±0.014	** 0.508 ± 0.008**	0.507±0.020
		Average	0.526±0.041	0.312±0.038	0.537±0.033	** 0.549 ± 0.058**	0.545±0.038
	MLP	REALDISP	0.598±0.009	0.546±0.035	0.608±0.012	** 0.620 ± 0.006**	0.613±0.006
		WEAR	0.517±0.011	0.411±0.051	0.524±0.012	0.533±0.006	**0.537±0.008**
		Average	0.557±0.041	0.479±0.095	0.566±0.042	** 0.576 ± 0.061**	0.575±0.038

The results show a consistent trend in favour of combining masked reconstruction with rotation-derived constraints, although the relative contribution of each term varies across datasets, sensor placements, and classifier heads. The reconstruction-only objective $L_{rec}$ remains a strong baseline in several configurations, particularly for ankle-mounted sensors. However, the variants including the Gram-based term are frequently among the best-performing alternatives. In particular, $L_{rec}+L_{gram}$ ranks first or second in 14 out of the 24 configurations, indicating that the temporal relational constraint provides a relevant contribution when added to masked reconstruction. The full objective $L_{rec}+L_{siam}+L_{gram}$ shows the most consistent ranking across the complete evaluation, appearing first or second in 21 out of the 24 configurations. While the best-performing variant varies in some individual cases, this ranking pattern suggests that combining the two rotation-derived constraints provides a stable formulation across different datasets, sensor placements, and classifier heads. The role of the Siamese-based term is more dependent on the sensor placement. The variant $L_{rec}+L_{siam}$ improves over $L_{rec}$ mainly for wrist-mounted sensors, whereas its effect is smaller or mixed for ankle-mounted sensors. In contrast, the unidirectional Siamese baseline alone is generally less competitive than the reconstruction-based variants, suggesting that the siamese alignment is more effective when used as an additional constraint on top of masked reconstruction rather than as a standalone objective. Overall, the ablation study supports the proposed design of combining reconstruction with rotation-derived supervision, while also showing that the contribution of each term is not uniform across all settings. The Gram-based term provides the clearest individual contribution among the added constraints, and the full objective offers the most consistent top-ranked behaviour across configurations, as reflected by the number of best and second-best results. This trend suggests that the interaction between independently masked views and the Gram-based loss may provide a complementary representation-level signal that improves downstream classification performance. Taken together, these results suggest that reconstruction, representation-level similarity encouraged by the Siamese loss, and temporal relational consistency promoted by the Gram loss provide complementary training signals for learning inertial representations.

Next, we analyze the misclassifications made by our framework in the frozen-encoder setting, where an MLP classification head is trained on top of encoder weights learned through the SSL task and then kept frozen. Table 3 shows the best- and worst-classified activities for the right wrist and left ankle across all datasets. We focus on these two sensor positions because they are available in all datasets. Across datasets, misclassifications are not arbitrary but concentrate in a small set of plausible alternatives, indicating that errors are dominated by location mismatch and within-family of activities ambiguity. Location mismatch is clear when the sensor is weakly coupled to the defining motion: for instance, at the left ankle in MHEALTH, frontal elevation of arms is the worst class and is primarily confused with sitting and relaxing, which is consistent with both activities producing limited lower-limb dynamics while the discriminative signal lies in the upper body. Within-family of activities ambiguity appears when classes share similar temporal structure and intensity at a given location: in WEAR, jogging (butt-kicks) is mainly confused with jogging, and at the left ankle jogging (sidesteps) is most confused with jogging (skipping), suggesting that the model reliably identifies the broader locomotion family but struggles to separate fine-grained variants from a single sensor. The same pattern is observed in REALDISP where the left-ankle jump up class is most often confused with jump side-ways, and in WEAR where stretching (shoulders) is frequently confused with stretching (lunging), both reflecting subtle inter-class differences that may require additional body locations or more context to resolve. Finally, low-dynamic activities tend to collapse into other posture-like movements: for the right wrist in MHEALTH, standing still is predominantly predicted as waist bends forward, reflecting the limited discriminative evidence provided by minimal motion, which leads to greater confusion with closely related low-activity patterns.

**Table 3:** Best- and worst-recognized activities for the right-wrist and left-ankle sensor positions across datasets using the MLP classification head, together with the dominant misclassification for each case (that is, the most frequently confused class). In the Main Confusion column, the number of confused windows is shown in parentheses. Pos denotes sensor position, Correct indicates the number of correct predictions, and Total represents the total number of instances for that activity.

Pos	Dataset	Activity	Correct	Total	Main Confusion
Right Wrist	MHEALTH	jump front & back (Best)	585	760	jogging (74)
		standing still (Worst)	68	2400	waist bends forward (892)
	PAMAP2	running (Best)	2954	3820	rope jumping (258)
		standing (Worst)	1708	7420	vacuum cleaning (1975)
	REALDISP	rowing (Best)	1728	2175	upper trunk & lower body opposite twist (300)
		shoulders low-amplitude rotation (Worst)	0	820	rowing (187)
	WEAR	jogging (butt-kicks) (Best)	4845	7710	jogging (1309)
		push-ups (complex) (Worst)	963	8350	sit-ups (complex) (1314)
Left Ankle	MHEALTH	lying down (Best)	1959	2400	sitting and relaxing (182)
		frontal elevation of arms (Worst)	82	2310	sitting and relaxing (569)
	PAMAP2	lying (Best)	5843	7550	ascending stairs (599)
		standing (Worst)	817	7420	sitting (1899)
	REALDISP	jump leg/arms open/closed (Best)	833	1320	lateral elevation of arms (99)
		jump up (Worst)	29	685	jump side-ways (234)
	WEAR	jogging (sidesteps) (Best)	6411	8890	jogging (skipping) (1207)
		stretching (shoulders) (Worst)	486	7720	stretching (lunging) (1540)

**Fig. 4:**
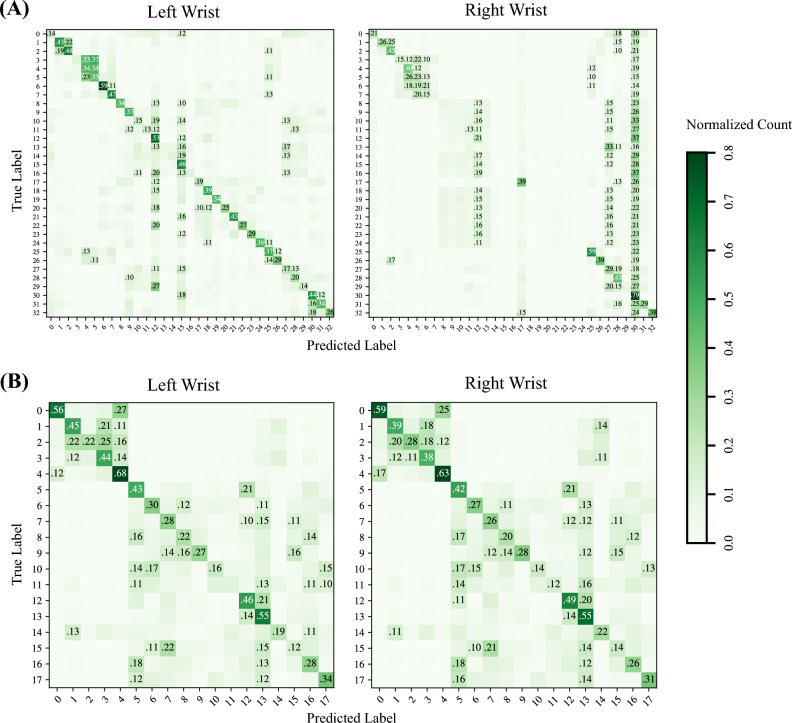
Confusion matrices for left and right wrist sensors. (**A**) REALDISP: while the left wrist maintains relatively distinct class separations, the right wrist shows predictions collapsing primarily into three classes (waist bending, knee bending, and rowing), particularly for activities beyond basic locomotion (classes 0–7). (**B**) WEAR: symmetric confusion patterns can be observed. Values below 10% are omitted for readability.

Next, we analyze performance across symmetric body parts for WEAR and REALDISP, which contain bilateral sensor placements. Both datasets showed symmetric performance for ankles, consistent with the bilateral locomotion patterns in their activity sets (available in the supplementary material, Appendix A, Table 2). For wrist-mounted sensors, the two datasets exhibited contrasting patterns. The WEAR dataset shows similar performance between left and right wrists, as expected given that most activities in this dataset involve symmetrical upper-body movements (e.g., push-ups, burpees, sit-ups). REALDISP, however, showed substantial asymmetry: the left wrist achieved better performance than the right wrist (see Fig. 4 (A)), with notably different confusion patterns. Examining the confusion matrices reveals that for classes 0–7 (walking, jogging, running, and jumping variants), both wrists show similar confusion patterns. However, for the right wrist, the majority of predictions for other activities collapse into three classes: waist bending, knee bending, and rowing. This suggests the right wrist signal becomes less discriminative for REALDISP’s more complex upper-body movements, particularly rotational and reaching exercises. One possible explanation is hand dominance: if participants were predominantly right-handed, the dominant hand may exhibit more variable movement execution across different activities, making patterns harder to distinguish. However, REALDISP does not report participant handedness, so we cannot verify this hypothesis. Alternative explanations include sensor placement differences or calibration artifacts specific to the right wrist sensors.

In addition to the confusion matrices, we conducted cross-position experiments on the wrist sensors for both REALDISP and WEAR. Specifically, we pretrained the model using our self-supervised objective on one wrist and then fine-tuned it on the opposite wrist, evaluating both right-to-left and left-to-right transfer. The results are reported in Table 4. For WEAR, the cross-position results indicate that representations learned from one wrist can transfer effectively to the opposite wrist. Transferring from the left wrist to the right wrist slightly improves performance compared with the same-position right-wrist baseline for both classifiers. Similarly, for the MLP classifier, right-to-left transfer also improves over the left-wrist baseline, while the Linear classifier shows only a small decrease. Overall, these results support the interpretation that both wrists capture comparable movement patterns in WEAR, which is consistent with the symmetry observed in the confusion matrices. In contrast, REALDISP shows a different transfer pattern. Pretraining on the right wrist and fine-tuning on the left wrist leads to a clear improvement over the left-wrist same-position baseline, reaching 0.646 and 0.661 for the Linear and MLP classifiers, respectively, compared with 0.583 and 0.613 when pretraining and fine-tuning on the left wrist. However, the opposite direction shows a strong degradation: left-to-right transfer achieves only 0.234 and 0.262, which is substantially lower than the right-wrist same-position baselines of 0.344 and 0.372. These results, together with the asymmetric pattern observed in Fig. 4, suggest that the relationship between the two wrist positions in REALDISP is more complex than in WEAR. While this asymmetry may be related to differences in movement execution across sides, we cannot rule out the influence of dataset-specific factors, such as sensor placement, calibration, or other acquisition-related effects.

**Table 4:** Cross-position wrist experiment between the left and right sides. Results are reported as macro F1-score, expressed as average ± standard deviation. During fine-tuning, all available training data were used. Models pretrained on one wrist were fine-tuned on the opposite wrist, considering both right-to-left and left-to-right transfer.

Dataset	Classifier	Right Wrist *→* Right Wrist	Right Wrist *→* Left Wrist	Left Wrist *→* Left Wrist	Left Wrist *→* Right Wrist
REALDISP	Linear	0.344±0.028	0.646±0.015	0.583±0.010	0.234±0.019
REALDISP	MLP	0.372±0.020	0.661±0.015	0.613±0.006	0.262±0.013
WEAR	Linear	0.475±0.020	0.471±0.016	0.507±0.020	0.524±0.009
WEAR	MLP	0.544±0.005	0.566±0.006	0.537±0.008	0.570±0.011

**Fig. 5:**
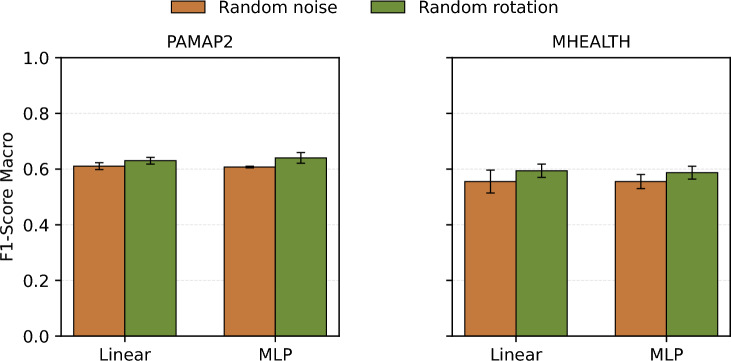
Comparison of random rotation and random noise as data augmentation techniques on right-wrist data from PAMAP2 and MHEALTH. The encoder is kept frozen, and both an MLP and a linear classifier are trained on top using all available training data.

Next, we perform an ablation study on the type of data augmentation used. The use of random rotation is motivated by previous studies in the field and by the geometric information introduced by this transformation. To further analyze the role of rotation within our framework, we compare it against additive Gaussian noise, which perturbs the signal by adding randomly sampled noise with the same shape as the input. Figure 5 reports the Macro F1-score obtained on the right-wrist sensor data from PAMAP2 and MHEALTH. Across the evaluated configurations, random rotation consistently outperforms additive Gaussian noise, with absolute improvements ranging from 2.0% to 3.9 % Macro F1 for both classifier heads and datasets. The average gain is 2.95 % for the linear classifier and 3.25% for the MLP. These results further support the use of rotation as a data augmentation strategy within the proposed framework.

**Fig. 6:**
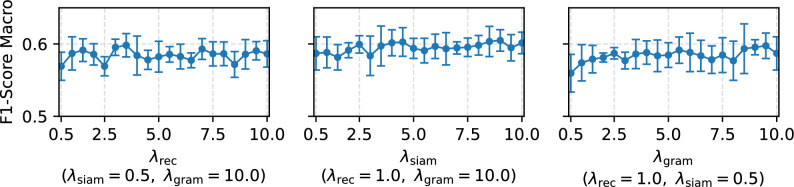
Weight sensitivity study for the self-supervised objective defined in Equation 13, conducted using the MHEALTH right-wrist data. Two loss weights are kept fixed at their original values, while the remaining weight is swept from 0.5 to 10.0 in increments of 0.5. Experiments follow the previous experimental protocol: after pre-training, the encoder is kept frozen and an MLP classification head is trained on top using all available training data.

**Fig. 7:**
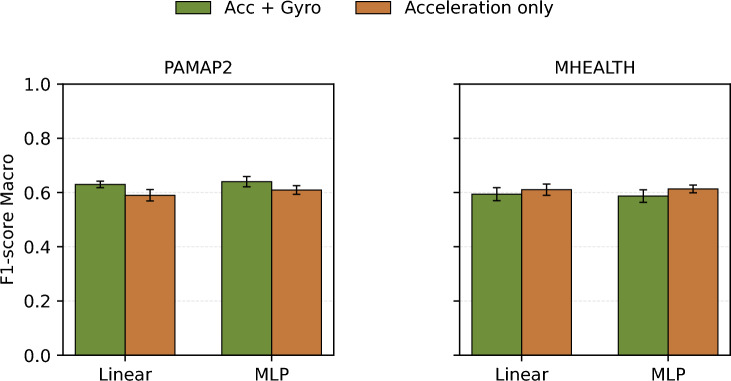
Signal ablation study. We compare the full pipeline using right-wrist acceleration and gyroscope signals, corresponding to the original implementation, against an acceleration-only configuration to isolate the effect of gyroscope data. In both settings, the encoder is first pretrained with the proposed self-supervised method. The pretrained encoder is then frozen, and either a linear or MLP classification head is fine-tuned using all available training data. Results are reported in terms of macro F1-score.

Next, we perform an ablation study on the weights of the self-supervised loss presented in Equation 13, using the MHEALTH right-wrist data due to the high computational cost of this analysis. Specifically, we keep two weights fixed to their original values and vary the remaining one from 0.5 to 10.0 in steps of 0.5. These limits correspond to the minimum and maximum weights used in the proposed loss function. Figure 6 reports the results in terms of macro F1-score. Overall, the results show that the method is reasonably stable across the explored weight ranges. Although some fluctuations are observed, there is no clear performance collapse or monotonic degradation when varying any individual weight. Interestingly, a slight upward trend can be observed when increasing $\lambda _{siam}$ and $\lambda _{gram}$, suggesting that assigning more weight to the Siamese and Gram-based objectives may provide a modest benefit in this setting. However, these gains are small and accompanied by local fluctuations, so this trend should be interpreted as indicative rather than conclusive. The original weights lie within a stable performance region, supporting their use as a balanced trade-off between the reconstruction, Siamese, and Gram-based terms. Therefore, while further tuning could provide minor improvements for specific settings, the selected configuration appears adequate for the experiments considered in this work. Finally, we perform an ablation study on the signals used during both pre-training and fine-tuning of the proposed framework. Specifically, we compare the original formulation, which uses both acceleration and gyroscope signals, against an acceleration-only configuration. The training follows the same protocol described above. First, the encoder is pretrained using the proposed self-supervised objective. Then, the encoder is frozen, and either a linear or MLP classification head is trained using the available activity labels. Figure 7 reports the macro F1-score obtained for the right-wrist sensor on PAMAP2 and MHEALTH. The effect of removing gyroscope data appears to be dataset-dependent, which may be related to differences in the activity composition of each benchmark. In PAMAP2, using both acceleration and gyroscope signals provides a small improvement over the acceleration-only setting. In contrast, in MHEALTH, acceleration-only inputs achieve better performance. Although no definitive conclusion can be drawn from these results, one possible explanation is that the discriminative value of gyroscope data depends on the extent to which the activities involve rotational dynamics. The MHEALTH activity set contains several activities that may be largely characterised by posture, acceleration magnitude, or gross movement dynamics, such as standing still, sitting, lying down, walking, climbing stairs, cycling, jogging, running, jumping, knees bending, and waist bends. Therefore, acceleration-only inputs may already provide sufficient information to discriminate many classes in this dataset. In contrast, PAMAP2 includes activities for which angular motion may provide complementary information to acceleration, such as Nordic walking, vacuum cleaning, ironing, and rope jumping. In these cases, gyroscope measurements may help capture rotational dynamics that are not fully represented by acceleration alone.

## Limitations and future works

Despite the encouraging results, this work still has some limitations. Although the proposed framework exploits rotation as a geometrically meaningful source of self-supervision, it does not yet fully capture all the information that rotations can provide about sensor orientation and movement structure. In particular, the current objective enforces similarity between rotated and original signals at the representation level, but does not explicitly model orientation-aware relationships or the geometric structure induced by successive rotations. This is particularly important in real-world HAR, where sensor orientation can vary considerably across users, recording sessions, wearing conditions, and device placements. Future work should therefore investigate how to extract richer information from rotation transformations, for example by incorporating explicit orientation reasoning, more principled geometric constraints in the latent space, or objectives that better capture the relationship between motion dynamics and sensor reference frames. Advancing in this direction could improve not only the quality and interpretability of the learned representations, but also the robustness and practical applicability of HAR systems beyond controlled benchmark settings.

## Conclusions

In this work, we presented a novel self-supervised pre-training approach for inertial-based human activity recognition that combines masked reconstruction with a Siamese-inspired objective and a time-based similarity metric to exploit the information provided by rotation transformations. Our approach is motivated by the fact that rotations are geometrically meaningful in IMU data: they correspond to changes in the sensor reference frame that preserve the underlying motion while redistributing the signal across axes. By encouraging consistency between the latent representations of rotated and original signals, the proposed method learns more informative representations for downstream HAR tasks. We evaluated the approach on four well-established HAR datasets, namely PAMAP2, MHEALTH, REALDISP, and WEAR, using subject-wise folds to account for inter-subject variability. All evaluations were performed under a single-sensor setup, considering four body positions: left ankle, right ankle, left wrist, and right wrist. Across these experimental settings, our method generally outperformed the baselines, yielding average gains of 1.4 percentage points with a linear head and 1.2 percentage points with an MLP head over the strongest competitor in terms of macro F1-score, when averaged across datasets and body positions. We further investigated the performance of the proposed method under varying amounts of labeled training data during the finetuning stage, showing that it remains competitive with a fully supervised baseline even when labeled data are limited. In addition, the ablation study provided a clearer understanding of the contribution of each component of the framework. Finally, the activity-wise and symmetry analyses offered further insight into the behaviour of the proposed method and helped to better characterise its strengths across activities and body locations.

## Supplementary Information

Supplementary Information.

## Data Availability

The datasets analysed during the current study are available in the UC Irvine Machine Learning Repository and the University of Siegen repository, Germany. The links to the datasets are: PAMAP2 (https://archive.ics.uci.edu/dataset/231/pamap2+physical+activity+monitoring), MHEALTH (https://archive.ics.uci.edu/dataset/319/mhealth+dataset), REALDISP (https://archive.ics.uci.edu/dataset/305/realdisp+activity+recognition+dataset), and WEAR (https://ubi29.informatik.uni-siegen.de/wear_dataset/).
