# Supplementary material for: Siamese masked reconstruction with temporal rotation consistency for inertial-based human activity recognition
Source: Sci Rep. 2026 Aug 14;16:25518. doi: 10.1038/s41598-026-64846-5 (PMC13476224; doi:10.1038/s41598-026-64846-5)
Supplement: Supplementary file 1 [file 41598_2026_64846_MOESM1_ESM.pdf]

Suplementary material

Francisco M. Calatrava-Nicolás[1,*], Todor Stoyanov[1], and Oscar Martinez Mozos[2,3]

[1]Department of Science and Technology, Örebro University, Örebro, 70182, Sweden
[2]Human Robotics, DFESTS, University of Alicante, Alicante, 03690, Spain
[3]MAD-Robotics, ETSIDI, Universidad Politécnica de Madrid, Madrid, 28012, Spain
[*]francisco.calatrava-nicolas@oru.se

ABSTRACT

Inertial-based Human Activity Recognition (HAR) aims at inferring activities performed by an individual from data on wearable inertial sensors. However, HAR often suffers from poor generalization to unseen users due to data heterogeneity across different individuals and conditions. While collecting and curating large labeled datasets could help alleviate this limitation, it is also costly. To reduce this gap without relying on extensive annotated data, we propose a self-supervised framework grounded in how IMU signals transform under rotation: applying a rotation matrix changes how motion projects onto the sensor axes without altering the underlying motion. A model trained to align representations of a signal and its rotated counterpart is therefore encouraged to capture motion content rather than sensor-frame patterns. We exploit this property by training a Siamese masked convolutional autoencoder that learns by reconstructing masked inputs while aligning representations of original signals and its rotated version, further regularized by a temporal consistency loss that enforces agreement between the temporal structure of original and rotated representations within each window. We evaluate on four public HAR benchmarks covering diverse scenarios, including activities of the daily living (ADL) and sports activities, using cross-subject evaluation, where our method yields improvements of +1.4 and +1.2 percentage points over the strongest baseline with linear and MLP probes respectively, averaged across datasets and sensor positions. We further show that our method obtains competitive results against the fully supervised baseline in low-data regimes. The code is available on https://github.com/FranciscoCalatrava/Rotation_Siamese_Masked.git

Appendix A: Dataset Details

This appendix describes the details of the used datasets such as the folds, the number of windows per folds, the activities, etc.

Table 1. Participant assignment per dataset and fold. Within each dataset, participant IDs are listed in ascending order for each fold.

Dataset	Fold 1	Fold 2	Fold 3	Fold 4	Fold 5
PAMAP2	(1,2)	(3,4)	(5,6)	(7,8)	(-,-)
MHEALTH	(1,2)	(3,4)	(5,6)	(7,8)	(9,10)
REALDISP	(1-4)	(5-8)	(9-12)	(13-16)	(17)
WEAR	(1–5)	(6–10)	(11–15)	(16–20)	(21,22)

Table 2. Activity label names for PAMAP2, MHEALTH, REALDISP, and WEAR datasets. Within each row, activities are listed in the exact class order used by the classifier output layer (from label 0 to $K-1$, where K is the number of activities)

Dataset	# Act.	Activity names
PAMAP2	12	lying; sitting; standing; walking; running; cycling; Nordic walking; ascending stairs; descending stairs; vacuum cleaning; ironing; rope jumping
MHEALTH	12	standing still; sitting and relaxing; lying down; walking; climbing stairs; waist bends forward; frontal elevation of arms; knees bending (crouching); cycling; jogging; running; jump front & back.

Dataset	# Act.	Activity names
REALDISP	33	walking; jogging; running; jump up; jump front & back; jump sideways; jump leg/arms open/closed; jump rope; trunk twist (arms outstretched); trunk twist (elbows bent); waist bends forward; waist rotation; waist bends (reach foot with opposite hand); reach heels backwards; lateral bend (left+right); lateral bend with arm up (left+right); repetitive forward stretching; upper trunk & lower body opposite twist; lateral elevation of arms; frontal elevation of arms; frontal hand claps; frontal crossing of arms; shoulders high-amplitude rotation; shoulders low-amplitude rotation; arms inner rotation; knees (alternating) to the breast; heels (alternating) to the backside; knees bending (crouching); knees (alternating) bending forward; rotation on the knees; rowing; elliptical bike; cycling.
WEAR	18	jogging; jogging (rotating arms); jogging (skipping); jogging (sidesteps); jogging (butt-kicks); stretching (triceps); stretching (lunging); stretching (shoulders); stretching (hamstrings); stretching (lumbar rotation); push-ups; push-ups (complex); sit-ups; sit-ups (complex); burpees; lunges; lunges (complex); bench-dips.

Appendix B: Network Details

The overall framework is illustrated in Figure 1 from the main paper and comprises four modules: an encoder $f(\cdot)$(Table 3), a decoder $d(\cdot)$, a projection head $\psi(\cdot)$ (Table 5), and a prediction head $\varphi(\cdot)$ (Table 6). In this section of the appendix, we describe the structure of the networks used. In addition to the networks used during the self-supervised step, we use both a linear classifier (Table 8) and a MLP classifier (Table 7) to evaluate the performance of the SSL approaches in the downstream classification task. Please note that the code is provided alongside the paper; for implementation details, it is preferable to consult the code directly.

Table 3. Encoder architecture. The encoder takes as input $x \in \mathbb{R}^{B \times C \times T}$, where B denotes the batch size, C the number of input channels, and T the window length. The window length T is kept constant across all evaluated datasets, whereas C varies depending on the dataset. Specifically, $C \in \{3,6\}$, depending on whether only tri-axial accelerometer data are available or both tri-axial accelerometer and gyroscope data are included.

**Block**	**Operation**	**Kernel/Stride/Pad**	**Channels**
Conv1	Conv1D	4/2/1	$C \to 32$
	ReLU	–	–
Conv2	Conv1D	4/2/1	$32 \to 64$
	ReLU	–	–
Conv3	Conv1D	3/1/1	$64 \to 64$
	ReLU	–	–
	Conv1D	3/1/1	$64 \to 128$
Residual1	ReLU	–	–
	Conv1D	1/1/0	$128 \to 64$
	Add skip	–	–
	ReLU	–	–
	Conv1D	3/1/1	$64 \to 128$
Residual2	ReLU	–	–
	Conv1D	1/1/0	$128 \to 64$
	Add skip	–	–
	ReLU	–	–
Conv4	Conv1D	1/1/0	$64 \to 64$
**Output**	–	–	$z \in \mathbb{R}^{B \times 64 \times T_{\text{latent}}}$

Table 4. Decoder architecture. The decoder takes as input the output of the encoder defined in Table 3. B denotes the batch size, C the number of input channels, and T the window length. The window length T is kept constant across all evaluated datasets, whereas C varies depending on the dataset. Specifically, $C \in \{3,6\}$, depending on whether only tri-axial accelerometer data are available or both tri-axial accelerometer and gyroscope data are included.

**Block**	**Operation**	**Kernel/Stride/Pad**	**Channels**
Deconv1	ConvTranspose1D	3/1/1	$64 \to 64$
	ReLU	–	–
	Conv1D	3/1/1	$64 \to 128$
Residual1	ReLU	–	–
	Conv1D	1/1/0	$128 \to 64$
	Add skip	–	–
	ReLU	–	–
	Conv1D	3/1/1	$64 \to 128$
Residual2	ReLU	–	–
	Conv1D	1/1/0	$128 \to 64$
	Add skip	–	–
Post-stack	ReLU	–	–
Deconv2	ConvTranspose1D	4/2/1	$64 \to 32$
	ReLU	–	–
Deconv3	ConvTranspose1D	3/1/1	$32 \to C$
Deconv4	ConvTranspose1D	4/2/1	$C \to C$
**Output**	–	–	$\hat{x} \in \mathbb{R}^{B \times C \times T}$

Table 5. Projector architecture. The input of the projector is the output of the encoder defined in Table 3.

Head	Operation	Kernel/Stride/Pad	Channels
Projector	Conv1D	1/1/0	$64 \rightarrow 128$
	BatchNorm1D	–	$128 \rightarrow 128$
	ReLU	–	$128 \rightarrow 128$
	Conv1D	1/1/0	$128 \rightarrow 128$

Table 6. Predictor architecture. The input of the predictor is the output of the projector defined in Table 5.

Head	Operation	Kernel/Stride/Pad	Channels
Predictor	Conv1D	1/1/0	$128 \rightarrow 256$
	BatchNorm1D	–	$256 \rightarrow 256$
	ReLU	–	$256 \rightarrow 256$
	Conv1D	1/1/0	$256 \rightarrow 128$

Table 7. MLP classifier head. It takes as input the output of the encoder presented in Table 3.

Block	Operation	Kernel/Stride/Pad	Dims
Pool	AdaptiveAvgPool1D(1)	–	$T \rightarrow 1$
Flat	Flatten (start_dim=1)	–	–
FC1	Linear + ReLU	–	$64 \rightarrow 128$
FC2	Linear	–	$128 \rightarrow Y$

Table 8. Linear classifier head. It takes as input the output of the encoder presented in Table 3.

Block	Operation	Kernel/Stride/Pad	Dims
Pool	AdaptiveAvgPool1D(1)	–	$T \rightarrow 1$
Flat	Flatten (start_dim=1)	–	–
FC1	Linear	–	$64 \rightarrow Y$
